# Development and Characterization of Weft-Knitted Fabrics of Naturally Occurring Polymer Fibers for Sustainable and Functional Textiles

**DOI:** 10.3390/polym13040665

**Published:** 2021-02-23

**Authors:** Marcela Ferrándiz, Eduardo Fages, Sandra Rojas-Lema, Juan Ivorra-Martinez, Jaume Gomez-Caturla, Sergio Torres-Giner

**Affiliations:** 1 Textile Industry Research Association (AITEX), Plaza Emilio Sala 1, E-03801 Alcoy, Spain; marcela@gmail.com; 2 Technological Institute of Materials (ITM), Universitat Politècnica de València (UPV), Plaza Ferrándiz y Carbonell 1, 03801 Alcoy, Spain; efages@aitex.es (E.F.); juaivmar@doctor.upv.es (J.I.-M.); jaugoca@epsa.upv.es (J.G.-C.); 3 Research Institute of Food Engineering for Development (IIAD), Universitat Politècnica de València (UPV), Camino de Vera s/n, 46022 Valencia, Spain

**Keywords:** natural fibers, soy protein, chitin, coir, comfort, functional textiles, Circular Bioeconomy

## Abstract

This study focuses on the potential uses in textiles of fibers of soy protein (SP) and chitin, which are naturally occurring polymers that can be obtained from agricultural and food processing by-products and wastes. The as-received natural fibers were first subjected to a three-step manufacturing process to develop yarns that were, thereafter, converted into fabrics by weft knitting. Different characterizations in terms of physical properties and comfort parameters were carried out on the natural fibers and compared to waste derived fibers of coir and also conventional cotton and cotton-based fibers, which are widely used in the textile industry. The evaluation of the geometry and mechanical properties revealed that both SP and chitin fibers showed similar fineness and tenacity values than cotton, whereas coir did not achieve the expected properties to develop fabrics. In relation to the moisture content, it was found that the SP fibers outperformed the other natural fibers, which could successfully avoid variations in the mechanical performance of their fabrics as well as impair the growth of microorganisms. In addition, the antimicrobial activity of the natural fibers was assessed against different bacteria and fungi that are typically found on the skin. The obtained results indicated that the fibers of chitin and also SP, being the latter functionalized with biocides during the fiber-formation process, showed a high antimicrobial activity. In particular, reductions of up to 100% and 60% were attained for the bacteria and fungi strains, respectively. Finally, textile comfort was evaluated on the weft-knitted fabrics of the chitin and SP fibers by means of thermal and tactile tests. The comfort analysis indicated that the thermal resistance of both fabrics was similar to that of cotton, whereas their air permeability was higher, particularly for chitin due to its higher fineness, which makes these natural fibers very promising for summer clothes. Both the SP and chitin fabrics also presented relatively similar values of fullness and softness than the pure cotton fabric in terms of body feeling and richness. However, the cotton/polyester fabric was the only one that achieved a good range for uses in winter-autumn cloths. Therefore, the results of this work demonstrate that non-conventional chitin and SP fibers can be considered as potential candidates to replace cotton fibers in fabrics for the textile industry due to their high comfort and improved sustainability. Furthermore, these natural fibers can also serve to develop novel functional textiles with antimicrobial properties.

## 1. Introduction

Over the last several years, valorization of natural resources has become an important topic. Therefore, the idea of using agricultural and food processing by-products [1], or even wastes of the agricultural and food industries can be very interesting due to it may contribute to the development of the Circular Bioeconomy [2]. In addition, it provides the possibility of develop fiber-based products with new characteristics and applications. More recently, fibrous porous media have also been elucidated with fractal models [3,4].

In this context, an interesting natural and renewable source for textiles fibers is represented by the antimicrobial polysaccharides. Among these, chitin offers, together with cellulose, show the highest worldwide availability. Chitin is a β-(1,4)-N-acetyl-D-glucosamine found in the shells of crabs, lobsters, and shrimps, which are marine fishery by-products. It is biodegradable and can be additionally blended with cellulose or silk [5]. Furthermore, chitin and chitosan, which is obtained from chitin deacetylation, are carbohydrates that exhibit several active and bioactive properties. For instance, some novel functional applications of chitin fibers are focused on the medical sector such as bandages, sutures, etc. since it shows non-toxicity to humans [6]. Chitin also offers antimicrobial protection due to its structure prevents the growth of microorganisms such as bacteria, fungi, and viruses [7,8,9]. However, despite some chitin fibers have been reported in literature, chitin is not widely used at industrial scale in textile applications. In particular, chitin has a semi-crystalline structure with highly extended hydrogen bonds, which cause some difficulty in its solubilization. In addition, its degree of acetylation (DA), which is normally 0.9, indicates the presence of some amino groups and it also affects its solubility. However, these groups are susceptible to modifications in order to improve this characteristic [10]. Therefore, artificially converting it to fibrous architectures has emerged as a novel approach to modulate their surface activity, structure, and molecular flexibility/rigidity for expanded textile applications. Therefore, various strategies have been adopted to fabricate fibers from chitin, including electrospinning as well as dry and wet spinning [11]. Fibers characteristics vary according to the solvent and carbohydrate contents, showing values of tenacity from ~1.59 to 3.2 g/d and elongation from ~3 to 20% [12].

Proteins represent another novel source for natural fibers derived from agricultural and industrial by-products and wastes [13]. Their properties and functionalities are greatly influenced by their amino acid sequences that initiate the self-assembly into higher order structures with unique physicochemical properties [14]. Among protein-based plants, soy proteins (SPs) are widely processed and consumed all over the world. In particular, different types of SP resins can obtained from soybean harvesting and processing as a by-product [15]. In this process, soy flour is purified to obtain soy protein powder. For that, soybean meal is first centrifuged followed by acidification and neutralization processes [16]. SPs can be classified into four categories, namely 2S, 7S, 11S, and 15S, according to their sedimentation coefficients, in which 7S and 11S surpass 80% of the total amount. β-conglycinin is the most prevalent 7S globulin and composed of three major subunits, namely α (~67 kDa), α’ (~71 kDa), and β (~50 kDa), jointed through noncovalent interactions. Native glycinin (11S globulin, hexamer) is made up of six subunits, owing a molecular weight (M_W_) of 300–380 kDa. These subunits are either acidic or basic and form a hollow cylinder by electrostatic interaction, hydrogen, and disulfide bonds [17]. The quaternary structure of SPs is susceptible to changes of external conditions such as pH, temperature, and ionic strength. Moreover, its high β-sheet content (≥40%) provides the peptides necessary for self-assembled fibrillation [18]. During fabrication of amyloid-like fibrils, proteins dissociate and are hydrolyzed into peptides, which then assemble into protofilaments that intertwine in the final stage. This sequential process can be achieved by heating the proteins in an acid environment [19,20,21], where conditions of 80–95 °C and pH 1.6–2 are commonly used. In general, SP fibers exhibit a worm-like and periodic structure, typically with diameter of a few nm and length of several µm. SP can be molded into a wide variety of shapes, which include fibers, films, hydrogels, nanofibers, and solid parts [22,23]. Moreover, SP can be mixed with other components in order to improve their properties or achieve new ones, such as antioxidant capacity or antimicrobial activity [24]. SP fibers also present a luxurious appearance, good comfort, good chromaticity, which makes them brilliant, and improves dyeing fastness compared to other protein-based fibers, such as silk [25].

Nevertheless, one of the main drawbacks of natural fibers is that they are prone to the biological attack of microorganisms and insects [26]. Microorganism growth depends on their chemical structure, specific surface area, thread thickness, and capacity to retain moisture, oxygen, and nutrients [27,28,29]. Some of the problems caused by microorganisms are odor emissions, discoloring, degradation, and risk of causing infections. In addition, microorganisms can release enzymes and other organic compounds, which can trigger degradation. Additionally, it is necessary to take into account that the microbial growth can result in a deterioration of the mechanical properties with a loss in tensile strength, elongation, and visual changes [29]. As a result, the use of antimicrobial materials has recently gained importance in a wide variety of applications related to medical clothes and implements, housing, decorative, construction as well as textiles [30]. In this regard, *Escherichia coli* (*E. coli*, gram-negative) and *Staphylococcus aureus* (*S. aureus*, gram-positive) are commonly used to test the antimicrobial properties since both are human pathogens that show high resistance to common antimicrobial agents [31,32]. Furthermore, *Candida albicans* (*C. albicans*), *Trichophyton mentagrophytes* (*T. mentagrophytes*), and *Epidermophyton stockdaleae* (*E. stockdaleae*) are among the fungi tested since they can be located on the skin as well as in mucous membranes and are responsible for systemic infections with high mortality rate [33,34]. All of these microorganism have been associated with several infections due to their growth on the surface of textile fabrics, especially in hospitals or health care institutions [35].

This study originally assesses the potential of natural fibers of chitin, SP, and coir, which can be obtained from agricultural and food wastes, to produce functional fabrics produced by weft knitting for uses in textile applications. To this end, it involves the physical characterization and antimicrobial properties of the natural fibers against the most commonly tested bacteria and fungi. Finally, the thermal and tactile comforts of the natural fiber fabrics were also determined and compared to those of cotton and cotton-based fibers. The tactile comfort was related to mechanical properties and the interaction of the clothes with the human body, while thermal comfort characterization involved thermal resistance, water vapor resistance, and air permeability properties.

## 2. Materials and Methods

### 2.1. Materials

Chitin fibers were supplied by Tec Service S.r.L. (Villorba, Italy), whereas the SP fibers were provided by Swicofil (Emmen, Switzerland). According to the supplier, SP fibers are functionalized with undisclosed antimicrobial agents during the spinning process, which can restrain the growth of colon bacillus, impetico bacteria, and sporothric. Coir fibers, obtained from Swarna Trades (Kochi, India), were also tested for comparison purposes. Table 1 summarizes the main physical properties of these natural fibers indicated by their suppliers.

### 2.2. Yarn Processing

The three types of natural fibers were selected for yarn processing and subsequent weaving. This process started with the opening of the fiber bales, which consists of the extraction of the pressed fiber from its packaging by an automatic machine UNIfloc A 12 from Rieter China Textile Instruments Co., Ltd. (Changzhou, China). The second step was the separation of the flock into homogenous small flakes of fibers to facilitate their disaggregation and individualization in subsequent processes. Finally, the third step was the carded process within the opening and cleaning of the fibers. This last operation completed the individualization of the fibers to obtain a regular tape, which was folded on a coil in a cylinder card. Thereafter, the resultant folded tape was stretched, in order to obtain a wick, which was subjected to a certain twist using a ring twisting machine form MEERA Industries limited (Gujarat, India) to obtain the yarn.

### 2.3. Fabric Manufacturing

**Figure 1:**
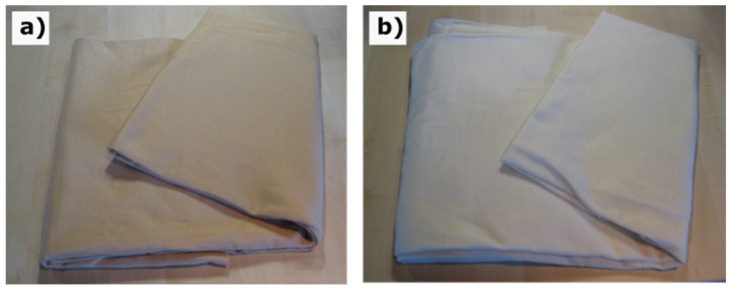
Weft-knitted fabrics of (**a**) soy protein (SP) (**b**) chitin fibers.

In order to perform the comfort tests, it was necessary to obtain the fabrics from the fibers. Knitting is, after weaving, the second most frequently used method to produce fabrics and its popularity has recently grown due to the adaptability of the new fibers, high versatility, and the consumer demand for stretchable, wrinkle resistant, and snug-fitting fabrics. Knitting to shape can be realized either by weft knitting or by warp knitting. In general, the weft-knitting process is more flexible due to the needle selection capability available and the variety of structural designs possible. In this technique, the same thread feeds an entire mesh pass, progressively feeding all the needles. Therefore, during this process, fabrics of several consecutive rows of intermeshed loops were made in a horizontal way from a single yarn and each consecutive rows of loop built upon the prior loops consecutively. To this end, the small-diameter circular machine Galan Ratera (Manresa, Spain) was used with a speed of 350 rpm. The resultant fabrics are shown in Figure 1.

### 2.4. Evaluation of Fiber Geometry and Mechanical Tests

The evaluation of fiber geometry, which is related to the fiber fineness, was carried out according to the classification of Sekhri [36], as shown in Table 2. 

The mechanical properties of the fibers were studied according to the UNE EN ISO 5079-96 and UNE EN ISO 1973:1996 analyzing tensile strength, elongation at break, tenacity, and fineness. The UNE 40152:1984 was followed for determining the length of the fibers. All the mechanical properties were obtained by averaging five experimental values, and all of the samples were stored and tested under room conditions.

### 2.5. Moisture Content Determination 

The ASTM D2495-07 standard test method, applied for moisture determination in cotton, was used to the study moisture content by oven drying. To this end, fibers were previously conditioned at 20 ± 2 °C and 65 ± 2% RH for 48 h, then, they were weighed and placed for drying at 105 ± 2 °C in an oven for 24 h. After this time, samples were cooled in a desiccator and weighed again. The moisture content was calculated following Equation (1),

(1) $$MC\;\left(\%\right)=\;\left[\left(M\;-\;D\right)/M\right]\times 100$$

where *MC* represents the moisture content (%), *M* is the mass of the specimen, and *D* is the oven-dry mass of the specimen. Samples were tested in triplicate.

### 2.6. Antimicrobial Tests

To ascertain the antibacterial and antifungal activities of the fibers, these were subjected to the presence of *E. coli* (ATCC 10536) and *S. aureus* (ATCC 6538) bacteria and *C. albicans* (CECT 1394), *T. mentagrophytes* (CECT 2958), and *E. stockdaleae* (CECT 2988) fungi. All bacteria and fungi were obtained from the Leibniz Institute DSMZ-German Collection of Microorganisms and Cell Cultures (Braunschweig, Germany). The evaluation of the antimicrobial efficacy of the fibers was carried out by following the principles of the AATCC Test Method 100-2004 standard. To this end, fiber samples of approximately 1 ± 0.1 g were introduced in the broth with the bacterial or fungal inoculum. Then, it was plated out by triplicate in agar plates after serial dilutions. Finally, samples were incubated for 18 h at 37 °C. After this time, the bacteria colony-forming units (CFU/mL) were determined for each plate. The microbial reduction or growth inhibition was quantified based on the number of CFU/mL identified after the incubation time for each type of fiber in relation to the number of bacteria initially counted. The same procedure was performed to analyze the antifungal activity.

### 2.7. Comfort Assessment

The comfort parameter is complex and subjective feature. It depends on both psychological and physiological perceptions of the body and the own fabric properties. It can be divided into tactile comfort and thermal comfort [37]. The comfort generated by a fabric depends, among other factors, on sensory parameters such as perception through the sense of touch, which is much more demanding and subjective than the rest. The aim of this measurements was to ascertain whether the comfort characteristics of the natural fiber fabrics could serve to replace textiles based on cotton. To this end, the KES-FB or Kawabata Evaluation System (Kato Tekko, Kyoto, Japan) was used to analyze the fabric low-stress mechanical and surface properties. It is based on different experimentation modulus: KES-FB1—automated module designed to measure the tension and shear properties of fabrics (tension energy, tension force, and stiffness and shear hysteresis), which applies opposing and parallel forces to the fabric until reach a maximum offset angle; KES-FB2—automated module to measure the flexion of a fabric or non-woven, where measures the force that a given fabric requires to be bended; KES-FB3—automated module to determine the compression properties of fabrics, in which a fabric of ~2 cm^2^ is subdued to a compress between two plates and increasing the pressure at a maximum value of 50 gf/cm^2^; and KES-FB4—automated module for measuring the surface of fabrics by determining the friction coefficient and geometric roughness with a displacement speed of the sample 1 mm/s [38]. Therefore, through the Kawabata Evaluation System, the three primary hand parameters shown in Table 3 were determined, which are habitually considered for analyzing the applications of fabrics in winter and autumn suiting [39].

The results obtained of tactile comfort in terms of primary hand values (PHVs) and total hand values (THVs) of the fabrics were qualified according to the evaluations described in Table 4 and Table 5, respectively [40].

The thermal comfort is defined as the condition in which the user feels satisfied with the thermal environment. Therefore, this concept is related to the fabric heat transmission behavior. In this analysis, three different tests were performed: Water vapor resistance, thermal resistance, and air permeability [41]. All these measurements were performed through Skin Model of Weiss Umwelttechnik (Lindenstruth, Germany), whose device allows quantifying the breathability of a given fabric and, therefore, the heat flow by varying the environmental conditions (%RH and temperature).

To compare the comfort results of the natural fabrics with conventional ones, the same tests were carried out on pure cotton and blends of cotton/polyester (35/65) and cotton/acrylic (40/60) fabrics obtained from Tela Prat S.A (Sabadell, Barcelona), which have the same stitch per weft. All the measurements were carried out at 65 ± 3% of RH at 20 ± 1 °C, averaging five experimental values.

### 2.8. Statistical Analysis

Mean values and standard deviations were calculated from the experimental data obtained by analysis of variance (ANOVA) with 95% confidence interval level (*p* ≤ 0.05). For this purpose, a multiple comparison test (Tukey) was followed using the software OriginPro8 (OriginLab Corporation, Northampton, MA, USA).

## 3. Results

### 3.1. Geometry and Mechanical Properties of the Natural Fibers

In the clothing industry, it is important to develop textiles with flexibility and elasticity that also offer functional properties that enjoy the growing demand for workwear and sportswear, thereby, improving the wearer’s comfort and protection [42,43]. Therefore, mechanical parameters of the fibers have a great importance both in terms of performance and also during the spinning process since they influence on the machine preparation. In Table 6, one can observe the different mechanical values of the natural fibers tested herein and obtained from the test. In this regard, it is important to consider that the characteristics of the natural fibers regarding mechanical properties and chemical composition can vary considerably according to the cultivation place, environmental conditions, extraction and processing methods, the part of the plant from which the fibers are obtained (roots, stem, leaves, etc.), maturity of the plant, among others [44,45]. Fineness, also called linear density, determines the quality and price of the fibers. In general terms, fibers are not constant or regular. Therefore, if a given fiber is thick, it will be stiff, firmer, and with wrinkle resistance. As opposite, if the fiber is fine, it will present softness, flexibility, and good fall [46]. In this sense, Hari [47] reported that fineness for cotton is between 1 and 3 dtex. According to the present results, the SP fiber value was within this range. On the contrary, the chitin fiber showed a higher value, being statistically different, but it was close to the upper limit. According to the evaluation reported in previous Table 2, both natural fibers can be classified as “semi-fine” fibers. These results in fineness are promising because SP and chitin are within the same range of cotton.

Fiber length is another very important feature due to it affects the processability that, in turn, also influences the physical properties. In the case of cotton fibers, their length ranges from 10 to 40 mm [48], being the most used worldwide those with a length between 20 and 35 mm [46]. Besides, it is known that the diameter of the cotton fiber, in most cases, is not more than 1/100 of the fiber length. The results indicate that the here-tested natural fibers are approximately 2 to 4 times longer than those of cotton, which is an indispensable factor to consider when choosing the spinning process and their application in textiles. In addition, another positive aspect of longer fibers is that they will require less twist than shorter fibers for yarn strengthen [47]. In this case, the SP fibers presented the highest value followed by coir and chitin, respectively.

The mechanical properties of the fibers also depend on their constitution, the amount of cellulose, and the crystallinity [49]. In this context, fibers obtained from fruits or seeds are habitually weaker than those obtained from steams of hemp or ramie. In particular, the higher tensile strength and modulus derive from their low microfibrillar angle and high cellulose content. However, it is also important to consider that the fibers strength can be more affected by their own defects than their structure [49]. In this context, SP and chitin, both natural fibers, showed similar values of elongation at break, that is, 16.21, and 17.87%, respectively. The ductility of these natural fibers is higher than that of cotton fibers, which has been reported to show an elongation-at-break value of approximately 7% [50]. For chitin, Fan et al. [7] indicated that blend fibers of chitin at 70 wt% with alginate yielded an elongation at break of 16.4%, which is close to the value attained in the present study. In addition, as it was mentioned above, the mechanical properties of the coir fibers were not analyzes due to their high brittleness, although other studies have reported values of elongation at break between 15 and 30% [51]. For instance, in the study performed by Sumi et al. [52], the elongation at break of coir fibers achieved a value of 16.67%, while Tomczak et al. [45] reported for 25-mm long fibers a value of approximately 25%.

In terms of tenacity, Jackman [53] described that the minimum value for most fiber fabrics is 22.12 cN/tex, though some fibers with lower values can be used in textiles due to they also show high elasticity and resilience. For instance, McKenna [50] reported that tenacity of cotton fiber is around 30 cN/tex, whereas Singh [54] indicated that the cotton fiber tenacity ranges between 25 to 40 cN/tex. Therefore, the previously reported values are close to the tenacity value of 32.12 cN/tex obtained herein for the SP fibers. The chitin fibers achieved a tenacity value of 24.73 cN/tex, which is similar to that reported previously by Pang et al. [55] for chitin/cellulose blended fibers, that is, 23 cN/tex. Therefore, one can consider that both SP and chitin fibers are above the minimum tenacity values required for being used in textiles. Considering that this property is related to their strength, which is the force per unit linear density necessary to break the fiber [36,56], these results make these natural fibers very promising to produce sustainable textiles.

### 3.2. Moisture Content of the Natural Fibers

Moisture content is one of the most important characteristics to be considered as it may affect the physical properties of fabrics [57]. For instance, it has been reported that moisture absorption for cotton can produce a significant increase in flexibility, toughness, elongation, length, tensile strength, and color, besides affecting the electrical properties [58,59]. Therefore, in textiles, controlling moisture content is necessary as an inadequate level of moisture can lead to fiber breakage, which causes a decrease in the quality of the fiber during harvesting and ginning. Additionally, moisture content of the fiber may also influence the fiber mass, which is an important factor in the textile market [60]. For instance, the increase in fiber strength in cotton is related to an increase in moisture content due to the formation of hydrogen bonding, while other fibers, such as rayon, become weaker when wet [36,56]. Another consequence of water absorption is swelling, which causes the increase in fiber diameter, and consequently, the contraction in the length of twisted ropes. In addition, a low moisture content will require excessive energy at the bale press, whereas a high moisture content can produce a deterioration of the fiber quality during storage due to microorganisms’ attack. Furthermore, the control of moisture is also important to facilitate cleaning. Therefore, a good control of this parameter can guarantee the adequate quality of the fibers all over the production and supplying chain [59].

**Figure 2:**
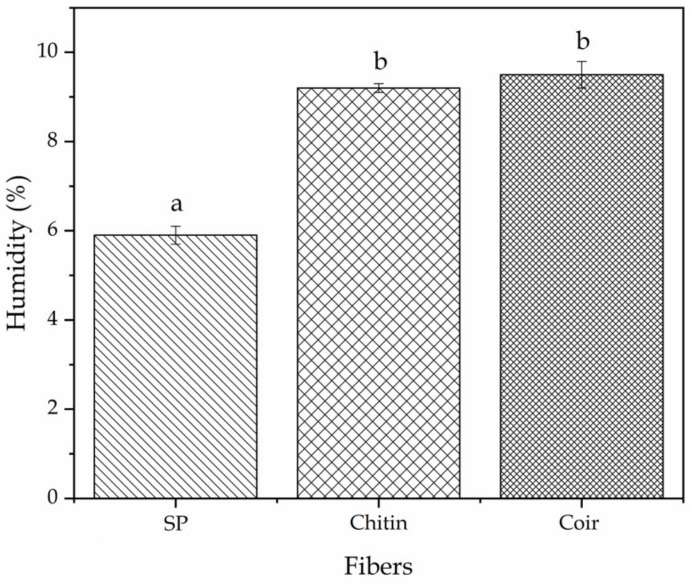
Percentage of moisture content (%) of soy protein (SP), chitin, and coir fibers. ^a,b^ Different letters indicate a significant difference among the samples (*p* < 0.05).

In textiles, this characteristic is normally expressed as the wet percentage material. One can observe in Figure 2 that the value obtained for SP fiber was 5.8%, whereas in the case of coir and chitin fibers, these values were 9.5%, and 9.2%, respectively. These moisture contents were also compared to those of cotton because this is the most popular natural fiber, particularly in clothing [48]. According to the study performed by Higgins et al. [61], the cotton fabric moisture content is around 8%, measured at 20 ± 2 °C with 65 ± 2% of RH. Similar results were attained for cotton fibers and cotton fabrics, that is, 8.5% in both cases [62]. According to Netravali [49], Tomczak et al. [45], and Mohanty et al. [63], the moisture content of coir fibers can be as high as 8%, while Cunha [64] indicated that chitin fiber is relatively hydrophobic. It is important to mention that, in general, plant fibers tend to absorb more quantity of water due to the presence of hydroxyl (-OH) groups in cellulose [48]. Therefore, the values reported, herein, agree with previous results and indicate that SP fiber absorbs less water amounts than the other natural fibers, including cotton. This is a positive factor since it favors its processability, cleaning, and mechanical performance stability. Besides, it is also important to note that the water content of SP fibers is higher than that of synthetic fibers based on polyesters, such as polyethylene terephthalate (PET), which shows a value of approximately 0.4% at 65% RH. However, excessively low moisture may cause static electricity due to friction [58] and it also produces dust and dirt attraction. For these reasons, synthetic fibers are habitually mixed with other hydrophilic material fibers [47].

### 3.3. Antimicrobial Activity of the Natural Fibers

**Figure 3:**
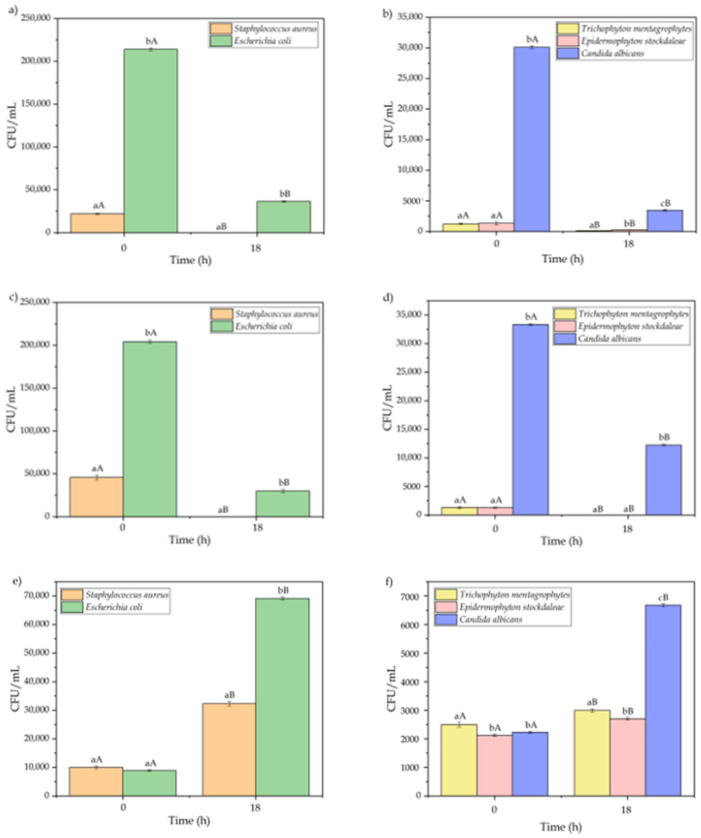
Antibacterial and antifungal activities in terms of colony-forming units (CFU/mL) of chitin fibers (**a**,**b**), soy protein (SP) fibers (**c**,**d**), and coir fibers (**e**,**f**). ^a–c^ Different letters in the same period for different samples indicate a significant difference (*p* < 0.05). ^A,B^ Different letter of the same sample in different periods indicate a significant difference (*p* < 0.05).

In the next step, the antimicrobial activity of the fibers was assessed against the *S. aureus* and *E. coli* bacteria and *T. mentagrophytes*, *E. stockdaleae*, and *C. albicans* fungi. The antimicrobial results of the natural fibers are gathered in Figure 3. One can observe in Figure 3a that chitin fibers showed a high antibacterial activity, especially against *E. coli*. In particular, after 18 h of contact, a reduction of 100% and 89.3% in the CFUs/mL of *S. aureus* and *E. coli* was respectively achieved. In this regard, Cheng et al. [28], reported that chitosan, a derivative from chitin, yielded a bacterial reduction for *S. aureus* of 98.57% and 98.59% with contact times of 30, and 50 min, respectively. In addition, for the same contact times, a reduction of 60.87% and 54.35% was observed for *E. coli*. Similarly, Qin et al. [65] developed chitosan fibers with silver sodium hydrogen zirconium phosphate by spinning solution, yielding a reduction of 98.60% in the growth of *S. aureus*. In another study, solution-spun blend fibers of chitin and cellulose were developed by Pang et al. [55]. In the fiber samples containing 6.46% of chitin, a reduction of 65.12% and 55.76% was obtained for *S. aureus* and *E. coli* growth. A high antibacterial activity was also observed by Cheah et al. [66], who developed a chitosan modified membrane with a quaternary amine (P-CS-GTMAC-A), having a reduction value of 76.7% for *E. coli*. The antibacterial activity of chitin and its derivatives involves electrostatic attraction. In the case of chitosan, for example, it has a positive charge that attracts bacterial cells charged negatively that produces the rupture of the bacterial cells and also causes the leak of the intracellular components [66,67]. In relation to the antifungal activity of chitin, shown in Figure 3b, one can observe that a reduction in the *T. mentagrophytes*, *E. stockdaleae*, and *C. albicans* strains of 88.8%, 79.1%, and 88.5% was respectively achieved after 18 h of contact. In the case of *C. albicans*, Qin et al. [65] observed a similar reduction trend by using chitosan fibers, reporting a reduction of 78.62% for this microorganism. Moreover, Surdu et al. [68] also analyzed the effect on the growth of *C. albicans* of cotton fabrics with different contents of chitosan, showing that the antimicrobial effect increased with the quantity of chitosan. In particular, for samples with 5 g/L of chitosan, the antimicrobial efficiency was nearly 83%, yielding an increase of 45% regarding the samples with the lowest chitosan value, that is, 1 g/L. Authors also analyzed the growth of other fungi of the Trichophyton family, that is, *T. interdigitale*, observing a reduction in the microbial growth of only 27% for cotton samples with 5 g/L of chitosan. This value is relatively lower compared with the one obtained in the present study, which can be related to the fact that chitosan was applied in the form of coating in the former study.

In relation to the SP fiber, its antibacterial activity against *S. aureus* and *E. coli* is shown in Figure 3c. Similar to the chitin fibers, at the beginning of the process, the bacterial growth was high, especially for *E. coli*. However, a reduction of 100% of the bacterial growth was observed after 18 h of contact. It is worth noting that SP alone has no antimicrobial effect and the present results differ from other studies regarding the use of SP fibers. For instance, soy protein isolated (SPI) film did not presented a decrease of the antimicrobial activity against Gram-positive *S. aureus* after 24 h of incubation [69]. In the same way, Xiao et al. [70] indicated that SPI did not presented antibacterial activity against both *S. aureus* and *E. coli*. On the other hand, in the study performed by Raeisi, et al. [71] it was observed for SPI/gelatin blends a slight decrease in the percentages of colonies (CFU) of 13% against *S. aureus*, while no colonies decrease was observed against *E. coli* after 24 h of incubation. Therefore, the antimicrobial performance of the SP fibers, reported herein, can be ascribed to the antimicrobial compounds added during the spinning process. These biocidal agents are mainly based on phenolics or acidic and bacteriocin since the functional groups present in the side chains of SP are known to alter their retention [72]. Some other examples of biocides added to the textiles include triclosan, silver, and quaternary ammonium compounds [73,74,75,76]. The mechanism used by antimicrobial textiles varies depending on their type, but in most cases, the main acting-mechanisms include preventing cell reproduction, damage on cell walls, protein denaturation, and enzyme blocking [77,78]. In relation to the antifungal activity of the SP fibers, which can be seen in Figure 3d, *T. mentagrophytes* and *E. stockdaleae* showed more than 1200 CFU/mL at the beginning of the test but both fungi were fully eliminated after 18 h of contact. In case of *C. albicans*, this reduction was of 63.3%, which still represents a good antifungal activity because it is above 50%. In comparison to other materials, Mishra et al. [79] reported the antibacterial activity of bamboo fibers, which presented lower antibacterial activities against *S. aureus* and *E. coli* with reduction values of 63.08%, and 68.44%, respectively. 

As opposed to the chitin and SP fibers, in the case of coir, one can observe that the number of microorganisms increased after 18 h of contact with the fibers. In particular, in Figure 3e one can observe that the number of CFU/mL at the beginning of the test was under 10,000 CFU/mL for both bacteria. However, it increased by approximately 3 and 7 times for *S. aureus* and *E. coli*, respectively. This behavior agrees with the results of the study performed by Chandy et al. [80], in which coir fibers did not present any inhibition zone against *E. coli*. These results mean that the fibers do not show any antibacterial activity. Similar results can be observed in Figure 3f for fungi, showing no evidence of antifungal activity after 18 h of contact. On the contrary, an increase in the number of CFU/mL was observed for all the fungi tested. In particular, the growth of *T. mentagrophytes*, *E. stockdaleae*, and *C. albicans* increased by 16.6%, 21.35%, and 66.6%, respectively. In this regard, Sumi et al. [52] reported that coir fibers do not present good antimicrobial properties against the *Aspergillus niger* fungus.

Therefore, one can conclude that both chitin and SP fibers present a high antibacterial activity against these two types of microorganisms. Additionally, the high increase in the number of bacteria and fungi observed for the coir fibers can be related to its high moisture content, which represents a more favorable medium for the development of microorganisms. However, it is also worth mentioning that the test conditions applied for all of the three types of fibers were the same, taking into account that microorganism growth also depends on temperature, pH, nutrients, oxygen and moisture content, etc. [81].

### 3.4. Comfort of the Natural Fiber Fabrics

Thermal comfort responds to the thermal environment that, in turns, depends on the temperature of the air and the room, the speed of the air and its humidity as well as on the type of clothing and the activity that is carried out. Therefore, water vapor resistance in fabrics is an important parameter due to the necessity that water vapor on the skin can pass through their pores, while an uncomfortable sensation can be produced if water vapor remains on the skin. In terms of thermal resistance, this parameter will depend on the type of fabric, its thickness, and bulk density, among others, whereas heat transmission involves the three different ways of transfer, that is, convection, conduction, and radiation. Furthermore, air permeability is related to the initial warm or cool feeling when the user wears clothes. If the airflow value is high, it means that the feeling of warm or cool will be more intense [82].

Table 7 shows the results of the thermal comfort analysis obtained through the skin model performed on the chitin and SP fabrics. These values were compared with fabrics of commercial cotton and blends of cotton with polyester and acrylic polymer to ascertain their application in textiles. Cotton is also a natural fiber, but it currently faces some environmental issues derived from the use of pesticides and insecticides to guarantee the good growth of the plants [83]. Furthermore, the coir fabric was discarded because as shown above, it did not achieve the expected properties.

One can observe that the water vapor resistance of the SP fabric was 5.01 m^2^·Pa/W, which was slightly but still significantly higher than that of cotton, that is, 4.82 m^2^·Pa/W, and also than the combinations of cotton with polyester and acrylic polymer, that is, 4.85, and 4.44 m^2^·Pa/W, respectively. In the case of the chitin fabric, the water vapor resistance value was 4.38 m^2^·Pa/W. These results are related to the moisture absorption capacity of each natural fiber, which agree with the moisture content shown in previous Figure 2, and also to its lower air permeability [82]. The SP fibers presented the lowest water absorption and showed the highest water vapor resistance. Moreover, according to the results obtained in this study, it can be concluded that the chitin fabric behaves similarly to that of cotton/acrylic, while the SP fabric would be more similar to that of neat cotton. These results indicate that the fabrics studied herein, being in a range of 4–5 m^2^·Pa/W, would have good breathability to water vapor. In comparison with previous works, Mishra et al. [79] reported that the water vapor resistance for cotton fabrics was 3.82 m2·Pa/W, being this value lower than that of the chitin and SP fabrics obtained in this study. This result can be explained by the fact that the previous cotton fabrics showed a fineness of 1.23 dtex, which is also lower than the present natural fibers. 

Regarding the thermal resistance of the fabrics, it can be observed that the SP one also presented the highest value, that is, 0.0463 m^2^·K/W. In the case of the chitin fabric, it showed a value of 0.0426 m^2^·K/W. However, it is important to mention that the thermal resistance of both fabrics was similar to those of cotton and cotton/polyester, which did not present significant differences, whereas the cotton-based fabric containing the acrylic polymer presented the lowest value. Thermal resistance is a very important parameter for fabrics intended for underwear applications, for example, in which cotton is mostly used. Therefore, in view of these results, it can be indicated that both the SP and chitin fabrics can be suitable for this type of application. For other types of textiles where the human body protection becomes more important due to climatic fluctuation, the SP fabric is the only one expected to provide the required properties.

Finally, the air permeability is the measure of air flow passed through a given area of a fabric, which is widely used in the textile industry for outdoor garment manufacturers to describe their functional performance. One can observe that the chitin fabric presented higher permeability than the pure cotton, showing a value of 2716 mm/s. In particular, this value was nearly 2 times higher than that of the cotton fabric, which was 1638 mm/s. Meanwhile, the SP fiber air permeability did not present a significant difference versus the cotton and cotton/polyester fabrics. The higher values attained herein can be related to the lower hairiness of these natural fibers due to they presented longer fiber lengths [82]. In the case of chitin, the high value of air permeability can be additionally related to high fineness of this natural fiber, which was 3.25 dtex, by which more air could penetrate through the fabric [41]. Therefore, considering the values obtained, herein, one can conclude that fibers of chitin and SP are more suitable to produce fabrics for summer clothing because in these fabrics the pass of the air is more favored.

Other important characteristic of fabrics is tactile sensation, which is crucial when determining the degree of comfort. The fabric hand properties allow knowing the performance of the material in contact with the skin, which depends on different factors such as fiber type, fabric structure, and, in some cases, the fabric finishing. For fabrics, there are two types of performance analysis. The first one is called utility performance and it is related to strength, color, and shrinkage, among others. The second one is quality performance, which is related to the appearance and comfort. It is important to consider that the second analysis is more difficult to conduct than the first one [84]. The assessment used to carry out the quality performance analysis is hand evaluation, being considered as a subjective method and known as Kawabata. Through this method the three PHVs, namely Koshi (stiffness), Numeri (smoothness), and Fukurami (fullness and softness), and also THVs were calculated in order to characterize the different fabrics. PHV is normally rated on a scale from 0 (weak) to 10 (strong), whereas THV is rated from 0 (not useful) to 5 (excellent) [85]. In Table 8 the results of the tactile comfort studied using Kawabata Evaluation System are gathered.

The fabric PHV concept covers the stiffness, fullness and softness of the fabric, and smoothness, which allows to quantify comfort based on the determination of the mechanical properties [86]. As seen in the Table 8, chitin presented values without significative differences, regarding cotton, and cotton/acrylic in Koshi parameter, which are related to the stiffness/elasticity when folded. In general, all samples values were ranged between 7.5 and 8.5, which indicates that they were high values. According to the reference used by Kawabata et al. [39], it can be considered that all of the samples presented a high-quality suiting for winter-autumn because their values are within the range of 4.5 ≤ HV ≤ 6.5. In the case of Fukurami, which indicates the fullness and softness in terms of body feeling and richness, it can be observed that both the SP and chitin fabrics showed relatively similar values than pure cotton. Nevertheless, it is important to consider that, within the current samples, the only value that was in the range of 5 ≤ HV ≤ 8 of high-quality suiting for winter-autumn was the cotton/polyester blend. Regarding Numeri, which determines the smoothness and it is ascribed to a smooth, flat, soft touch fabric, both chitin and SP fabrics showed lower values, that is, 4.06 and 6.15, respectively, than the pure cotton, which presented the highest value, that is, 8.56. However, the values attained for chitin fibers is in the range of 6 ≤ HV ≤ 8, which is considered as high-quality suiting for winter-autumn. Furthermore, the THV was also determined since this parameter is used to indicate the quality of the fabric for a specified end use and it gives a general idea of a fabric hand [87,88]. Therefore, the higher this value, the better the fabric touch and the more comfortable is the textile. The neat cotton fabric showed the highest value, that is, a THV of 3.72. As indicated in the table, it is considered as good, being relative similar to that reported by Behera [82], that is, 3.01. While, chitin presented no significant difference compared to cotton.

The results also indicated that the cotton/polyester fabric presents a good value, whereas in the case of SP and chitin their values are in the range that corresponds to average. The fabric with the lowest THV was attained for the cotton/acrylic combination, with a value of 2.72, although it could be still considered as average for this type of application. However, it must be necessary a THV ≥ 4 for winter-autumn and a THV ≥ 3.5 for midsummer, according to the criteria for an ideal fabric [39]. Based on this evaluation, only pure cotton and the blend of cotton/polyester fulfill this requirement for midsummer with values of 3.72, and 3.60, respectively. As reported by Sun et al. [89], the PHVs for Koshi, Numeri, and Fukurami and THV were 4.78, 0.05, 3.04, and 2.03, respectively. Therefore, it can be said that the stiffness and softness values were within the good range, however, the smoothness value was low. Thus, THV was also low and the fabric can be considered weak for winter-autumn clothes. In the study carried out by Verdu et al. [90], the behavior of the cotton/polyester blend in proportions of 35/65 showed values of PHV for Koshi, Numeri, and Fukurami and THV of 5.8, 4.6, 4.4, and 3.1, respectively. All comfort values were within the range of average, which indicates that the fabric can be potentially applied for winter clothes, while the THV parameter indicated that the fabric has good quality. From the above, one can consider that the SP fabric and, more importantly, the chitin fabric presented Koshi (stiffness), Fukurami (fullness and softness), and total hand values (THV) close to those of cotton fabrics, which opens up their potential applications in sustainable textiles.

## 4. Conclusions

Over the last years, new chemical and synthetic fibers have been developed to fulfill different technical requirements of the textile industry. These fibers result from chemical processes applied to natural fibers or, more habitually, derive from synthetic polymers. However, synthetic fibers show several sustainability issues due to the polymers used for their manufacturing derives from petroleum, which is not a renewable source, and the resultant textiles are not biodegradable or compostable, that is, they do not disintegrate in natural environments or in controlled compost conditions, respectively. In this regard, the valorization of wastes or processing by-products of the agricultural and food industries can potentially increase the economic value of natural fibers and also provide enhanced environmental benefits that improve the overall sustainability of the textiles. The present study reported on the use of chitin and SP fibers as an environmentally friendly alternative to chemical and synthetic fibers, whereas fabrics of both fibers were also considered as potential candidates to replace cotton. In terms of mechanical properties, both natural fibers presented values similar to cotton for fineness and tenacity. Furthermore, the chitin and SP fibers were longer than common cotton fibers, which is something desirable in textiles since their yarns will be less prone to twist when knitted. Moreover, in the case of SP, these fibers showed the lowest water absorption value, which is a positive aspect to improve the mechanical stability and reduce microbial growth. The antimicrobial tests also demonstrated that the chitin and SP fibers are very promising to avoid the growth of bacteria and fungi. In the case of chitin, its antimicrobial properties are ascribed to the interaction between positively charged biopolymer molecules and negatively charged microbial cell membranes. For SP, these fibers were functionalized by the manufacturer with microbial resistant elements integrated in the fiber molecule chains during the spinning process. The thermal comfort analysis also revealed that thermal resistance of the chitin and SP fibers had similar values than cotton whereas, regarding air permeability, both fabrics showed higher values than the cotton fabric. The lowest value was observed for chitin due to its higher fineness, indicating that these novel fabrics allow the air pass more easily through the fabric and, thus, can be potentially considered for summer clothes. For water vapor, the SP fabric presented higher resistance than chitin, which is related to the low moisture content of these fibers. In terms of PHV, the stiffness for the SP and chitin fabrics turned out to be high, being more than the average, however softness and fullness had similar values to that of cotton. Alternatively, smoothness presented more variability among the tested fabrics, showing cotton the highest value. In addition, the THV parameter showed adequate values for SP and chitin, being in the range of average, while the cotton/polyester fabric showed the only value that corresponded to the good range to meet the requirements for winter-autumn cloths. 

## Figures and Tables

**Table 1:** Main physical properties of soy protein (SP), chitin, and coir fibers.

Properties	SP Fiber	Chitin Fiber	Coir Fiber
Density (g/cm^3^)	1.29	1.43	1.40
Fiber length (mm)	38	37–39	50–150
Linear density (dtex)	0.9–3.0	1.57–1.77	-
Tenacity (g/tex)	38.8–40.8 *	23.4–27.5	10.0
Elongation at break (%)	18–21 *	18–22	30
Moisture content (%)	8.6	< 20	8

* dry properties.

**Table 2:** Fineness classification of fibers.

Type of Fiber	Fiber Fineness (dtex)
Thick fibers	>7
Semi-fine fibers	7–2.4
Fine fibers	2.4–1
Microfibers	1–0.3
Super microfibers	< 0.3

**Table 3:** Primary hand parameters for fabrics following the Kawabata Evaluation System.

Test	Primary Hand Parameters
Koshi	Stiffness and stiffness/elasticity when folded
Numeri	Smoothness
Fukurami	Fullness and softness

**Table 4:** Primary Hand Values (PHVs) for fabrics according to the feeling grade.

PHV	Feeling Grade
10	Extremely strong
9	Strong
8	-
7	-
6	-
5	Average
4	-
3	-
2	Weaker
1	Extremely weak
0	No feeling

**Table 5:** Total Hand Values (THVs) for fabrics.

THV	Grade
5	Excellent
4	Good
3	Average
2	Fair
1	Poor
0	Not useful

**Table 6:** Mechanical properties of soy protein (SP), chitin, and coir fibers.

Properties	Fineness (dtex)	Length (mm)	Tensile Strength (cN)	Elongation at Break (%)	Tenacity (cN/tex)
SP fiber	2.45 ± 0.88 ^a^	108.12 ± 0.74 ^a^	7.87 ± 0.63 ^a^	16.21 ± 0.98 ^a^	32.12 ± 1.10 ^a^
Chitin fiber	3.25 ± 1.21 ^b^	87.26 ± 0.96 ^b^	8.04 ± 0.74 ^b^	17.87 ± 0.93 ^a^	24.73 ± 0.79 ^b^
Coir fiber *	-	98.40	-	30.00	9.80

* Obtained from the technical datasheet of the product; ^a,b^ Different letters in the same column indicate a significant difference among the samples (*p* < 0.05).

**Table 7:** The results obtained through skin model for fabrics of soy protein (SP), chitin, cotton, cotton-based fibers.

Fabrics	Water Vapor Resistance (m^2^·Pa/W)	Thermal Resistance (m^2^·K/W)	Air Permeability (mm/s)
Chitin	4.38 ± 0.11 ^a^	0.0426 ± 0.0023 ^a^	2716 ± 40 ^a^
SP	5.01 ± 0.11 ^b^	0.0463 ± 0.0017 ^a^	1792 ± 70 ^b^
Cotton	4.82 ± 0.07 ^c^	0.0425 ± 0.0019 ^a^	1638 ± 150 ^c^
Cotton/Polyester (65/35)	4.85 ± 0.03 ^c^	0.0417 ± 0.0010 ^a^	1829 ± 90 ^b^
Cotton/Acrylic (60/40)	4.44 ± 0.05 ^a^	0.0379 ± 0.0008 ^b^	1614 ± 120 ^c^

^a–c^ Different letters in the same column indicate a significant difference among the samples (*p* < 0.05).

**Table 8:** Results obtained through Kawabata of the primary and total hand values (PHVs and THVs) of men’s winter suit fabrics of soy protein (SP), chitin, cotton, and cotton-based fibers.

Fabric	PHVs						THVs	
	Koshi		Fukurami		Numeri			
	Value	Grade	Value	Grade	Value	Grade	Value	Grade
SP	7.71 ± 0.08 ^a^	Strong	4.70 ± 0.21 ^a^	Average	4.06 ± 0.05 ^a^	Average	3.23 ± 0.06 ^a^	Average
Chitin	8.40 ± 0.04 ^b^	Strong	3.94 ± 0.15 ^b^	Average	6.15 ± 0.04 ^b^	Average	3.42 ± 0.07 ^b,c^	Average
Cotton	7.90 ± 0.05 ^b,c^	Strong	4.44 ± 0.11 ^a,b^	Average	8.56 ± 0.07 ^c^	Strong	3.72 ± 0.09 ^c^	Good
Cotton/polyester (35/65)	7.44 ± 0.03 ^c^	Strong	5.43 ± 0.09 ^c^	Average	6.81 ± 0.12 ^d^	Average	3.60 ± 0.07 ^c^	Good
Cotton/acrylic (40/60)	8.16 ± 0.10 ^b^	Strong	1.54 ± 0.19 ^d^	Weak	3.77 ± 0.03 ^e^	Weak	2.72 ± 0.13 ^d^	Average

^a–e^ Different letters in the same column indicate a significant difference among the samples (*p* < 0.05).

## Data Availability

Data is contained within the article.

## References

[1] Jiang B., Wang L., Wang M., Wu S., Wang X., Li D., Liu C., Feng Z., Chi Y. (2021). Direct Separation and Purification of α-Lactalbumin from Cow Milk Whey by Aqueous Two-phase Flotation of Thermo-sensitive Polymer/Phosphate. J. Sci. Food Agric..

[2] Jiang B., Na J., Wang L., Li D., Liu C., Feng Z. (2019). Reutilization of food waste: One-step extration, purification and characterization of ovalbumin from salted egg white by aqueous two-phase flotation. Foods.

[3] Liang M., Fu C., Xiao B., Luo L., Wang Z. (2019). A fractal study for the effective electrolyte diffusion through charged porous media. Int. J. Heat Mass Transf..

[4] Xiao B., Huang Q., Wang Y., Chen H., Chen X., Long G. (2020). A fractal model for capillary flow through a single tortuous capillary with roughened surfaces in fibrous porous media. Fractals.

[5] Rinaudo M. (2006). Chitin and chitosan: Properties and applications. Prog. Polym. Sci..

[6] Lee J.-W. (2008). Antimicrobial Agents and Applications on Polymeric Materials. Text. Color. Finish..

[7] Fan L., Du Y., Zhang B., Yang J., Cai J., Zhang L., Zhou J. (2005). Preparation and properties of alginate/water-soluble chitin blend fibers. J. Macromol. Sci. Part A Pure Appl. Chem..

[8] Hsieh S.H., Huang Z., Huang Z., Tseng Z. (2004). Antimicrobial and physical properties of woolen fabrics cured with citric acid and chitosan. J. Appl. Polym. Sci..

[9] Pan Y., Huang X., Shi X., Zhan Y., Fan G., Pan S., Tian J., Deng H., Du Y. (2015). Antimicrobial application of nanofibrous mats self-assembled with quaternized chitosan and soy protein isolate. Carbohydr. Polym..

[10] Pillai C., Paul W., Sharma C.P. (2009). Chitin and chitosan polymers: Chemistry, solubility and fiber formation. Prog. Polym. Sci..

[11] Ifuku S., Saimoto H. (2012). Chitin nanofibers: Preparations, modifications, and applications. Nanoscale.

[12] Dutta P.K., Ravikumar M., Dutta J. (2002). Chitin and chitosan for versatile applications. J. Macromol. Sci. Part C Polym. Rev..

[13] Mohammad F. (2016). Sustainable natural fibres from animals, plants and agroindustrial wastes—An overview. Sustainable Fibres for Fashion Industry.

[14] Yigit S., Dinjaski N., Kaplan D.L. (2016). Fibrous proteins: At the crossroads of genetic engineering and biotechnological applications. Biotechnol. Bioeng..

[15] Burgos N., Valdés A., Jiménez A. (2016). Valorization of agricultural wastes for the production of protein-based biopolymers. J. Renew. Mater..

[16] Leceta I., Etxabide A., Cabezudo S., de la Caba K., Guerrero P. (2014). Bio-based films prepared with by-products and wastes: Environmental assessment. J. Clean. Prod..

[17] Nishinari K., Fang Y., Guo S., Phillips G.O. (2014). Soy proteins: A review on composition, aggregation and emulsification. Food Hydrocoll..

[18] Herrero A.M., Jiménez-Colmenero F., Carmona P. (2009). Elucidation of structural changes in soy protein isolate upon heating by Raman spectroscopy. Int. J. Food Sci. Technol..

[19] Tang C.-H., Wang C.-S. (2010). Formation and Characterization of Amyloid-like Fibrils from Soy β-Conglycinin and Glycinin. J. Agric. Food Chem..

[20] Tang C.-H., Wang S.-S., Huang Q. (2012). Improvement of heat-induced fibril assembly of soy β-conglycinin (7S Globulins) at pH 2.0 through electrostatic screening. Food Res. Int..

[21] Xia W., Zhang H., Chen J., Hu H., Rasulov F., Bi D., Huang X., Pan S. (2017). Formation of amyloid fibrils from soy protein hydrolysate: Effects of selective proteolysis on β-conglycinin. Food Res. Int..

[22] Cho S.Y., Park J.-W., Batt H.P., Thomas R.L. (2007). Edible films made from membrane processed soy protein concentrates. LWT Food Sci. Technol..

[23] Wang Q., Liu W., Tian B., Li D., Liu C., Jiang B., Feng Z. (2020). Preparation and characterization of coating based on protein nanofibers and polyphenol and application for salted duck egg yolks. Foods.

[24] Jiang B., Wang X., Wang L., Wu S., Li D., Liu C., Feng Z. (2020). Fabrication and Characterization of a Microemulsion Stabilized by Integrated Phosvitin and Gallic Acid. J. Agric. Food Chem..

[25] Yi-You L. (2004). The Soybean Protein Fibre-A Healthy & Comfortable Fibre for the 21^st^ Century. Fibres Text. East. Eur..

[26] Pekhtasheva E., Neverov A., Kubica S., Zaikov G. (2012). Biodegradation and biodeterioration of some natural polymers. Polym. Res. J..

[27] Fernandes J.C., Tavaria F.K., Fonseca S.C., Ramos Ó.S., Pintado M.E., Malcata F.X. (2010). In vitro screening for anti-microbial activity of chitosans and chitooligosaccharides, aiming at potential uses in functional textiles. J. Microbiol. Biotechnol..

[28] Cheng X., Ma K., Li R., Ren X., Huang T. (2014). Antimicrobial coating of modified chitosan onto cotton fabrics. Appl. Surf. Sci..

[29] Szostak-Kotowa J. (2004). Biodeterioration of textiles. Int. Biodeterior. Biodegrad..

[30] Zhao Y., Xu Z., Lin T. (2016). Barrier textiles for protection against microbes. Antimicrobial Textiles.

[31] Han S., Yang Y. (2005). Antimicrobial activity of wool fabric treated with curcumin. Dyes Pigment..

[32] Esquenazi D., Wigg M.D., Miranda M.M., Rodrigues H.M., Tostes J.B., Rozental S., Da Silva A.J., Alviano C.S. (2002). Antimicrobial and antiviral activities of polyphenolics from Cocos nucifera Linn.(Palmae) husk fiber extract. Res. Microbiol..

[33] Tayel A.A., Moussa S., Wael F., Knittel D., Opwis K., Schollmeyer E. (2010). Anticandidal action of fungal chitosan against Candida albicans. Int. J. Biol. Macromol..

[34] Ryan K.J., Ray C.G. (2003). Sherris Medical Microbiology.

[35] Ristić T., Zemljič L.F., Novak M., Kunčič M.K., Sonjak S., Cimerman N.G., Strnad S. (2011). Antimicrobial efficiency of functionalized cellulose fibres as potential medical textiles. Sci. Against Microb. Pathog. Commun. Curr. Res. Technol. Adv..

[36] Sekhri S. (2019). Textbook of Fabric Science: Fundamentals to Finishing.

[37] Gandhi K. (2019). Woven Textiles: Principles, Technologies and Applications.

[38] Schindler W.D., Hauser P.J., Schindler W.D., Hauser P.J. (2004). 3—Softening finishes. Chemical Finishing of Textiles.

[39] Kawabata S., Niwa M., Yamashita Y. (2002). Recent developments in the evaluation technology of fiber and textiles: Toward the engineered design of textile performance. J. Appl. Polym. Sci..

[40] Behery H. (2005). Effect of Mechanical and Physical Properties on Fabric Hand.

[41] Raj S., Sreenivasan S. (2009). Total wear comfort index as an objective parameter for characterization of overall wearability of cotton fabrics. J. Eng. Fibers Fabr..

[42] Paul R. (2014). Functional Finishes for Textiles: Improving Comfort, Performance and Protection.

[43] Shishoo R. (2005). Textiles in Sport.

[44] Ramesh M., Palanikumar K., Reddy K.H. (2017). Plant fibre based bio-composites: Sustainable and renewable green materials. Renew. Sustain. Energy Rev..

[45] Tomczak F., Sydenstricker T.H.D., Satyanarayana K.G. (2007). Studies on lignocellulosic fibers of Brazil. Part II: Morphology and properties of Brazilian coconut fibers. Compos. Part A Appl. Sci. Manuf..

[46] Alonso Felipe J.V. (2015). Manual Control de Calidad en Productos Textiles y Afines.

[47] Hari P.K. (2020). Types and properties of fibres and yarns used in weaving. Woven Textiles.

[48] Yu C. (2015). Natural textile fibres: Vegetable fibres. Textiles and Fashion.

[49] Netravali A.N. (2005). Biodegradable Natural Fiber Composites.

[50] McKenna H.A., Hearle J.W.S., O’Hear N. (2004). Ropemaking materials. Handbook of Fibre Rope Technology.

[51] Li X., Tabil L.G., Panigrahi S. (2007). Chemical treatments of natural fiber for use in natural fiber-reinforced composites: A review. J. Polym. Environ..

[52] Sumi S., Unnikrishnan N., Mathew L. (2017). Surface modification of coir fibers for extended hydrophobicity and antimicrobial property for possible geotextile application. J. Nat. Fibers.

[53] Jackman D.R., Dixon M.K. (2003). The Guide to Textiles for Interiors.

[54] Singh J.P., Verma S., Singh J.P., Verma S. (2017). 3—Raw materials for terry fabrics. Woven Terry Fabrics.

[55] Pang F.J., He C.J., Wang Q.R. (2003). Preparation and properties of cellulose/chitin blend fiber. J. Appl. Polym. Sci..

[56] Bunsell A.R. (2018). Handbook of Properties of Textile and Technical Fibres.

[57] Montalvo J., Von Hoven T. (2008). Review of standard test methods for moisture in lint cotton. J. Cotton Sci..

[58] ASTM International (2019). Standard Test Method for Moisture in Cotton by Oven-Drying1.

[59] Delhom C., Rodgers J. Cotton Moisture—Its Importante, Measurements and Impacts. Proceedings of the 33rd International Cotton Conference Bremen.

[60] Hu J., Li Y., Yeung K.-W., Wong A.S., Xu W. (2005). Moisture management tester: A method to characterize fabric liquid moisture management properties. Text. Res. J..

[61] Higgins L., Anand S., Hall M., Holmes D. (2003). Effect of tumble-drying on selected properties of knitted and woven cotton fabrics: Part I: Experimental overview and the relationship between temperature setting, time in the dryer and moisture content. J. Text. Inst..

[62] General Administration of Quality Supervision, Inspection and Quarantine of the People’s Republic of China (2008). Conventional Moisture Regains of Textiles. GB 9994-2008. National Standard of the People’s Republic of China.

[63] Mohanty A., Misra M., Drzal L.T. (2001). Surface modifications of natural fibers and performance of the resulting biocomposites: An overview. Compos. Interfaces.

[64] Cunha A.G., Gandini A. (2010). Turning polysaccharides into hydrophobic materials: A critical review. Part 2. Hemicelluloses, chitin/chitosan, starch, pectin and alginates. Cellulose.

[65] Qin Y., Zhu C., Chen J., Zhong J. (2007). Preparation and characterization of silver containing chitosan fibers. J. Appl. Polym. Sci..

[66] Cheah W.Y., Show P.-L., Ng I.-S., Lin G.-Y., Chiu C.-Y., Chang Y.-K. (2019). Antibacterial activity of quaternized chitosan modified nanofiber membrane. Int. J. Biol. Macromol..

[67] Jiao Y., Niu L.N., Ma S., Li J., Tay F.R., Chen J.H. (2017). Quaternary ammonium-based biomedical materials: State-of-the-art, toxicological aspects and antimicrobial resistance. Prog. Polym. Sci..

[68] Surdu L., Stelescu M.D., Iordache O., Manaila E., Craciun G., Alexandrescu L., Christian Dinca L. (2016). The improvement of the resistance to Candida Albicans and Trichophyton interdigitale of some cotton textile materials by treating with oxygen plasma and chitosan. J. Text. Inst..

[69] Li S., Donner E., Xiao H., Thompson M., Zhang Y., Rempel C., Liu Q. (2016). Preparation and characterization of soy protein films with a durable water resistance-adjustable and antimicrobial surface. Mater. Sci. Eng. C.

[70] Xiao Y., Liu Y., Kang S., Wang K., Xu H. (2020). Development and evaluation of soy protein isolate-based antibacterial nanocomposite films containing cellulose nanocrystals and zinc oxide nanoparticles. Food Hydrocoll..

[71] Raeisi M., Mohammadi M.A., Coban O.E., Ramezani S., Ghorbani M., Tabibiazar M., Noori S.M.A. (2020). Physicochemical and antibacterial effect of Soy Protein Isolate/Gelatin electrospun nanofibres incorporated with Zataria multiflora and Cinnamon zeylanicum essential oils. J. Food Meas. Charact..

[72] Rani S., Kumar R. (2019). A Review on Material and Antimicrobial Properties of Soy Protein Isolate Film. J. Polym. Environ..

[73] Cerkez I., Kocer H.B., Worley S., Broughton R., Huang T. (2012). Multifunctional cotton fabric: Antimicrobial and durable press. J. Appl. Polym. Sci..

[74] Simoncic B., Tomsic B. (2010). Structures of novel antimicrobial agents for textiles-a review. Text. Res. J..

[75] Gao Y., Cranston R. (2008). Recent advances in antimicrobial treatments of textiles. Text. Res. J..

[76] Le Ouay B., Stellacci F. (2015). Antibacterial activity of silver nanoparticles: A surface science insight. Nano Today.

[77] Vigo T.L. (2001). Antimicrobial Polymers and Fibers: Retrospective and Prospective.

[78] Schindler W.D., Hauser P.J. (2004). Chemical Finishing of Textiles.

[79] Mishra R., Behera B., Pada Pal B. (2012). Novelty of bamboo fabric. J. Text. Inst..

[80] Chandy M., Kumar K.S. (2015). Characterisation of Silver Deposited Coir Fibers by Magnetron Sputtering. CORD.

[81] Abatenh E., Gizaw B., Tsegaye Z., Wassie M. (2017). The role of microorganisms in bioremediation—A review. Open J. Environ. Biol..

[82] Behera B. (2007). Comfort and handle behaviour of linen-blended fabrics. AUTEX Res. J..

[83] Shabbir M. (2019). Textiles and Clothing: Environmental Concerns and Solutions.

[84] Raheel M. (2017). Modern Textile Characterization Methods.

[85] Varghese N., Thilagavathi G. (2014). Handle and comfort characteristics of cotton core spun lycra and polyester/lycra fabrics for application as blouse. J. Text. Appar. Technol. Manag..

[86] Sun D., Stylios G.K. (2012). Cotton fabric mechanical properties affected by post-finishing processes. Fibers Polym..

[87] Jariyapunya N., Musilová B., Koldinská M. (2016). Evaluating the Influence of Fiber Composition and Structure of Knitting Fabrics on Total Hand Value (THV). Proc. Appl. Mech. Mater..

[88] Ji D.S., Lee J.J. (2016). Mechanical properties and hand evaluation of hemp woven fabrics treated with liquid ammonia. Fibers Polym..

[89] Sun D., Stylios G. (2005). Investigating the plasma modification of natural fiber fabrics-the effect on fabric surface and mechanical properties. Text. Res. J..

[90] Verdu P., Rego J.M., Nieto J., Blanes M. (2009). Comfort analysis of woven cotton/polyester fabrics modified with a new elastic fiber, part 1 preliminary analysis of comfort and mechanical properties. Text. Res. J..

